# Preparation of Tin Oxide Quantum Dots in Aqueous Solution and Applications in Semiconductor Gas Sensors

**DOI:** 10.3390/nano9020240

**Published:** 2019-02-11

**Authors:** Jianqiao Liu, Weiting Xue, Guohua Jin, Zhaoxia Zhai, Jiarong Lv, Wusong Hong, Yuzhen Chen

**Affiliations:** 1 College of Information Science and Technology, Dalian Maritime University, Dalian 116026, China; jqliu@dlmu.edu.cn (J.L.); jessxwt@sina.com (W.X.); jingh@dlmu.edu.cn (G.J.); jiaronglv@outlook.com (J.Lv.); hongwusong@outlook.com (W.H.); 2 Department of Material Science and Engineering, Dalian Maritime University, Dalian 116026, China; cyz7817@dlmu.edu.cn

**Keywords:** tin oxide, quantum dot, aqueous solution, semiconductor, gas sensor

## Abstract

Tin oxide quantum dots (QDs) were prepared in aqueous solution from the precursor of tin dichloride via a simple process of hydrolysis and oxidation. The average grain size of QDs was 1.9 nm. The hydrothermal treatment was used to control the average grain size, which increased to 2.7 and 4.0 nm when the operating temperatures of 125 and 225 °C were employed, respectively. The X-ray photoelectron spectroscopy (XPS) spectrum and X-ray diffraction analysis (XRD) pattern confirmed a rutile SnO_2_ system for the QDs. A band gap of 3.66 eV was evaluated from the UV-VIS absorption spectrum. A fluorescence emission peak was observed at a wavelength of 300 nm, and the response was quenched by the high concentration of QDs in the aqueous solution. The current-voltage (I-V) correlation inferred that grain boundaries had the electrical characteristics of the Schottky barrier. The response of the QD thin film to H_2_ gas revealed its potential application in semiconductor gas sensors.

## 1. Introduction

The semiconductor quantum dot (QD) is a very small three-dimensional system with dimensions of several nanometers. It is constituted by a few hundred atoms, much fewer than the atom number of a bulk system. Because of the small size of QDs, the energy states for electrons, holes, and excitons are discrete series, revealing the effect of quantum confinement. They bring unique characteristics that are valuable for producing advantageous electronic devices. Thus, in recent decades, QDs have attracted a lot of attention. For example, Alberto prepared PbS QDs for photoconductors, in which the control of surface chemistry offered a direct approach for the tuning of charge carrier dynamics based on strongly coupled QD solids [1]. Ternary alloy PbCdS QDs were explored as photosensitizers for quantum dot sensitized solar cells [2]. CdSe QDs were used as ultrasensitive sensors for Pb^2+^ heavy metal ions [3] and fluorescent nanomarkers for diesel oil [4]. Colloidal QDs of PbS, ZnO, and WO_3_ were good candidates for novel semiconductor gas sensors [5,6,7,8,9]. Even though the semiconductor QDs of CdSe, PbS, and CdS showed perspectives in the practical applications, their toxicities have to be considered. The elements of cadmium, lead, and telluride have been proved to be harmful to many kinds of living things, especially after their accumulation through the food web [10].

Tin oxide (SnO_2_) is a classical semiconductor, which has a wide direct band gap of 3.6 eV. It holds the advantages of good chemical stability, non-toxicity, and low cost. Recently, dozens of works have been published to report the applications of SnO_2_ QDs in novel electronic devices of gas sensors [11,12,13,14,15,16,17], photocatalysts [18,19], solar cells [20,21], and lithium-ion batteries [22,23]. Many techniques have been developed to prepare QD elements and devices, such as molecular beam epitaxy, metal-organic chemical vapor deposition, and some chemical routes [24]. However, these methods relied on complex facilities, or included organic compounds, which may bring potential toxicity to human beings or the environment. For example, SnO_2_ QDs were synthesized via a solvothermal route, in which organic reagents of oleic acid, oleylamine, and toluene were used [25,26]. Bathula prepared SnO_2_ QDs from the precursor of tin chloride pentahydrate with the aid of hydrazine and ethanol [27]. The consumption of toxic organics in the QD fabrication process not only put employees in danger but also increased the cost in environmental remediation by factories. Therefore, a simple and cheap method to prepared SnO_2_ QDs is expected.

In the present work, SnO_2_ QDs were prepared in aqueous solution via a simple route of hydrolysis and oxidation of SnCl_2_·2H_2_O, with the catalytic of CH_4_N_2_S. The hydrothermal treatment was introduced to control the grain growth of QDs. The characteristics of composition, morphology, grain size, photonic adsorption, fluorescence spectrum, and electrical property were concluded for the prepared QDs.

## 2. Materials and Methods 

SnCl_2_·2H_2_O of 0.01 mol and CH_4_N_2_S of 0.001 mol were dissolved into deionized water of 50 ml by a magnet stirring apparatus in order to acquire a solution, which was stirred for 24 h at 25 °C. The starting material of SnCl_2_·2H_2_O was transformed to SnO_2_ QDs after hydrolysis and oxidation, which were promoted by the existence of CH_4_N_2_S, as shown in Equation (1) and Equation (2). It was proved that this process was spontaneous but the resultant was not stable [28,29]. CH_4_N_2_S acted as stabilizer and accelerator because of the tautomerism between thiourea and isothiourea in the acidic solution [30,31]. It therefore consumed the HCl and accelerated the ongoing of Equation (1) as well as the formation of SnO_2_ QDs.

SnCl_2_ + 2H_2_O→Sn(OH)_2_ + 2HCl (1)

2Sn(OH)_2_ + O_2_→2SnO_2_ + 2H_2_O (2)

The obtained SnO_2_ QD solution was tagged as QD0, and it was put into the polyphenylene autoclave for hydrothermal treatment at 125 and 225 °C for 2 h, with QD125 and QD225 tags for the corresponding samples, respectively. The QD solutions were dried to obtain powders for compositional and optical characterizations. The QD0 solution was deposited on alumina substrates with silver electrodes, which were described in previous work [12]. The deposition process of QD thin film comprised a spin-coating at 1000 rpm and a subsequent heat treatment at 150 °C for 10 min. The deposition was repeated five times, and the obtained thin film was used for characterization of electrical and gas-sensing properties.

The morphology of SnO_2_ QDs were observed by transmission electron microscopy (TEM, JEM-3200FS, JEOL, Tokyo, Japan). The QD size distribution was obtained by dynamic light scattering (DLS, Zetasizer Nano ZS 90, Malvern panalytical Ltd., Malvern, UK). The dried powders were prepared for X-ray diffraction analysis (XRD, D/MAX-Ultima, Rigaku, Tokyo, Japan), X-ray photoelectron spectroscopy (XPS, ESCALAB 250 XI, ThermoFisher Scientific, Waltham, MA, USA), and ultraviolet–visible spectroscopy (UV-VIS, UH4150 Spectrophotometer, Hitachi, Tokyo, Japan). The fluorescence spectra of the QD0 solution and its diluted one were collected by a fluorescence spectrometer (FLS-980, Edinburgh Instruments, Edinburgh, UK). The electrical property and gas-sensing characteristics of QD0 thin films were measured by Keithley 2450 (Tektronix, Beaverton, OR, USA). The sensor response (*S*) was defined as the ratio of the thin film resistance in air (*R_a_*) to the ratio in the reducing gas (*R_g_*), as *S* = *R_a_*/*R_g_*. H_2_ was used as the representative of reducing target gases in order to check the gas-sensing characteristics of SnO_2_ QDs.

## 3. Results

**Figure 1:**
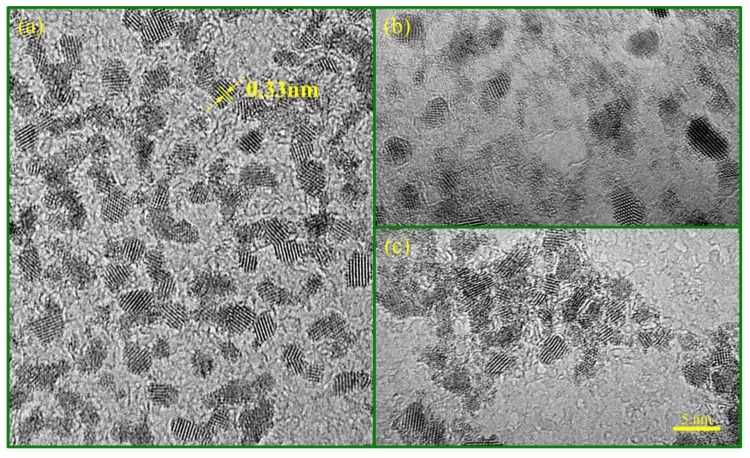
Transmission electron microscope morphologies of quantum dot (QD) samples: (**a**) QD0, (**b**) QD125, and (**c**) QD225.

Figure 1 illustrates the morphologies of SnO_2_ QDs from TEM observation, which shows the average grain sizes of 1.9 ± 0.2, 2.7 ± 0.4, and 3.9 ± 0.8 nm for QD0, QD125, and QD225 samples, respectively. The grains appeared to be uniform in size. QD0 and QD125 samples dispersed well in the aqueous solution, while a little aggregation was found in the QD225 sample. The characteristic spacing of 0.33 nm was observed, corresponding to the (110) planes of the rutile phase of SnO_2_.

**Figure 2:**
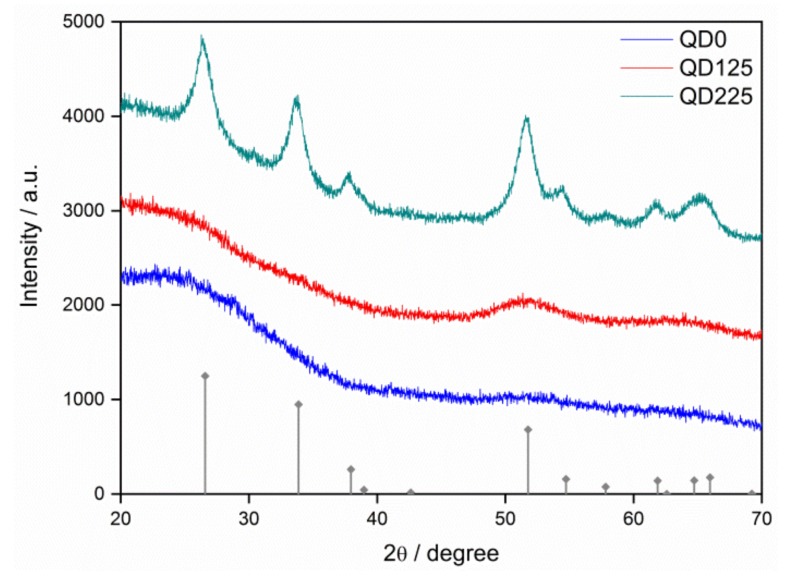
X-ray diffraction patterns of QD0, QD125, and QD225 samples with the standard pattern of SnO_2_ semiconductors.

The XRD patterns of QD0, QD125, and QD225 samples are revealed in Figure 2, in which the standard pattern of SnO_2_ peaks [32] was also involved. An extremely weak peak was observed for the (211) plane of the QD0 sample. After the hydrothermal treatment at 125 °C, the grain growth happened as the obvious (211) peak appeared. The whole SnO_2_ peak pattern was found when the temperature of hydrothermal treatment increased to 225 °C, as shown by the QD225 sample, which showed a complete crystal structure of the rutile SnO_2_ system. The crystallite size of each sample was calculated by the Scherrer’s formula based on the (211) peak. The QD125 and QD225 samples had crystallite sizes of 2.8 nm and 4.2 nm, respectively, while the crystallite size was less than 2.0 nm in the QD0 sample. The results were in agreement with the TEM observation.

**Figure 3:**
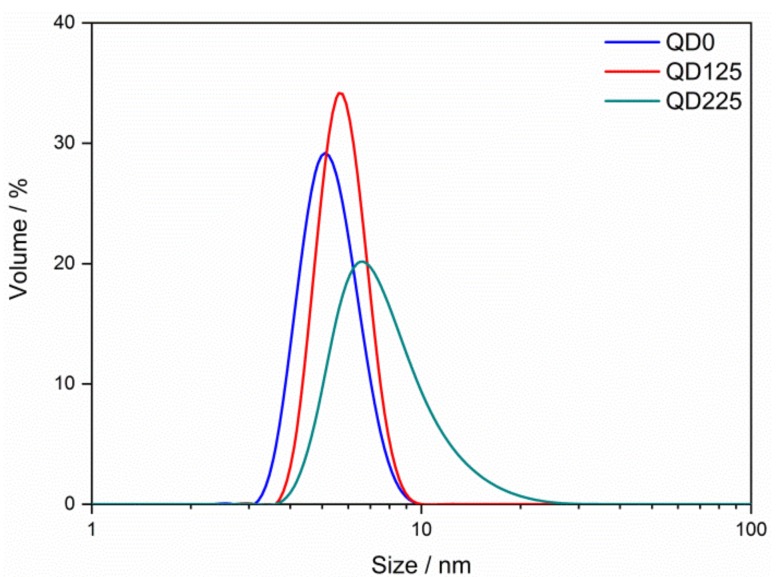
Size distributions of QD0, QD125, and QD225 samples in aqueous solution.

Figure 3 shows the size distributions of QD0, QD125, and QD225 samples in aqueous solution. The peaks appeared at 4.8, 5.6, and 6.5 nm, shifting to larger sizes with the incremental temperature of the hydrothermal process. The average grain sizes were 5.3 ± 1.1, 5.8 ± 0.9, and 8.0 ± 5.9 nm, respectively, and the results were larger than the ones from TEM observation and the XRD calculation. The results of TEM, XRD, and DLS showed that hydrothermal treatment is an effective way to control the grain size of SnO_2_ QDs. The grains grew up in the aqueous solution by combining tiny crystallites of SnO_2_, and the higher temperature of hydrothermal treatment provided more energy for combination. Therefore, it was possible to prepare desired QDs with controllable grain sizes by adjusting the temperature and time of hydrothermal treatment.

**Figure 4:**
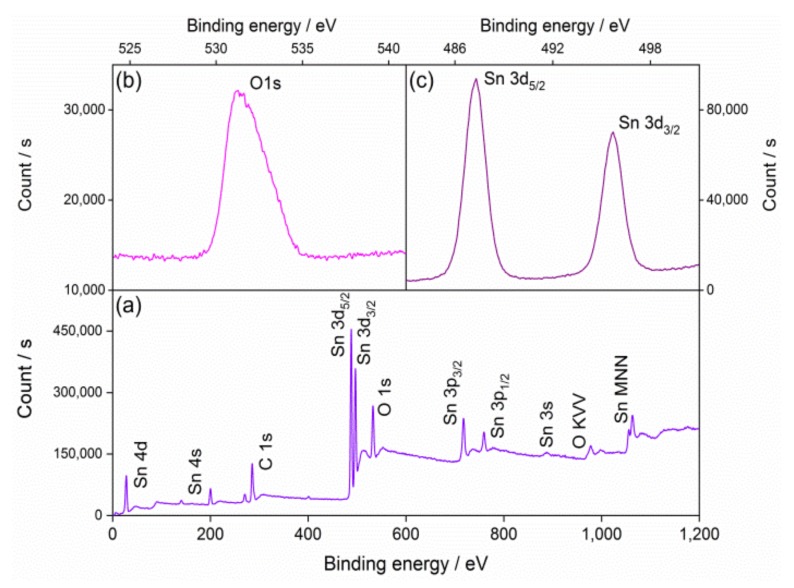
X-ray photoelectron spectrum of the QD0 sample: (**a**) Survey and high-solution peaks of (**b**) O 1s and (**c**) Sn 3d.

Figure 4a shows the survey XPS spectrum of the QD0 sample, which performed the existence of C, O, and Sn elements. Two peaks of 487.3 and 495.6 eV were observed in the high solution pattern of Sn 3d, corresponding to Sn 3d_3/2_ and Sn 3d_5/2_, as shown in Figure 4c. Meanwhile, the peak of O 1s appeared at 531.5 eV, as shown in Figure 4b. The results coincided with the standard pattern from the rutile SnO_2_ system [33,34].

**Figure 5:**
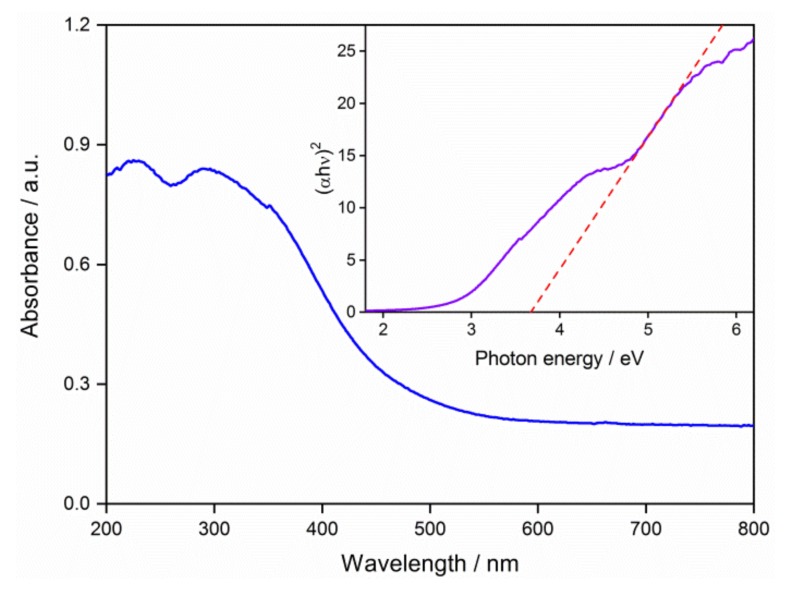
Ultraviolet-visible absorption spectrum of the QD0 sample and the evaluation of band gap (*E_g_*).

The UV-VIS absorption spectrum of the QD0 sample is shown in Figure 5, which shows two absorption peaks. The spectrum was transformed and plotted in the coordinates of (*αhν*)^2^ against *hν* in order to evaluate the band gap (*E_g_*). The *E_g_* was calculated to be 3.66 eV, with a slight increase compared to 3.6 eV of the bulk SnO_2_ material. The *E_g_* increase of SnO_2_ QDs was also concluded in other works [13,25,35]. However, the evaluated *E_g_* was smaller than the ones in the previous reports for QDs with similar sizes. The defects in the crystal lattice, such as oxygen vacancies, were potentially responsible for the slight enhancement of *E_g_*.

**Figure 6:**
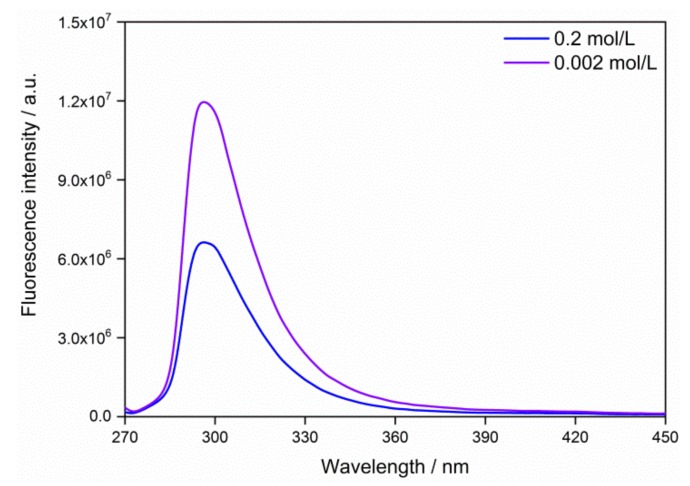
Fluorescence spectra of the QD0 sample with concentration of 0.2 and 0.002 mol/L.

The fluorescence spectra of the QD0 sample with the concentration of 0.2 and 0.002 mol/L are shown in Figure 6. The emission peaks at 300 nm were in agreement with the previous report [36]. The fluorescence quenching was observed when the concentration was as high as 0.2 mol/L. In this case, the fluorescence emission was inhibited by the nearby grains, which adsorbed the energy of electron transition from conduction band to valence band. It was ascribed to the self-quenching of fluorescence, which is the strong coupling of ground state fluorescence agents creating a stable, non-fluorescent complex [37]. The fluorescence self-quenching was driven by hydrophobic effects or π-π stacking of fluorescence agents at high concentrations [38].

**Figure 7:**
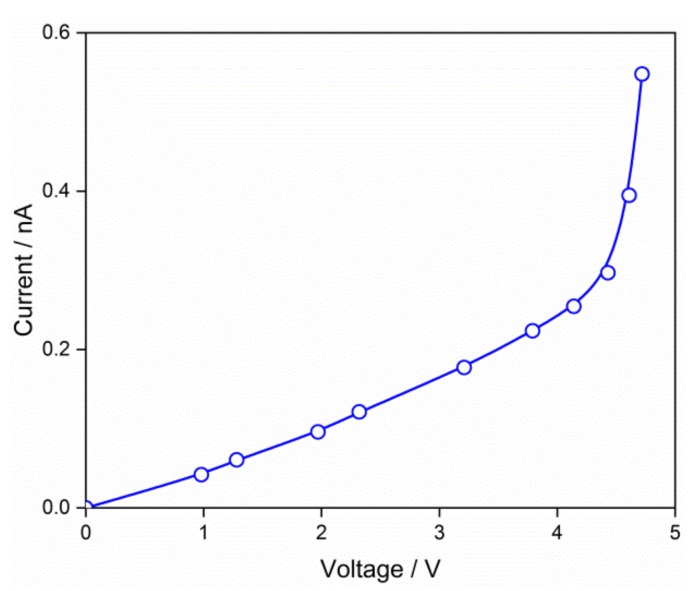
Voltage-current characteristics of the thin film prepared from the QD0 solution.

Figure 7 shows the voltage-current characteristics of the thin film, which was prepared from the QD0 solution. An approximately linear curve was observed between 0 and 4 V. A sharp increase took place when the voltage increased to 4–5 V. In this case, the depletion layer disappeared under the effect of an external biased electric field. The tendency of the curve was similar to the nanocrystalline SnO_2_ thin films with Schottky barrier at grain boundaries [39], revealing that the electrical properties of the QD thin film were also determined by the depletion layer.

**Figure 8:**
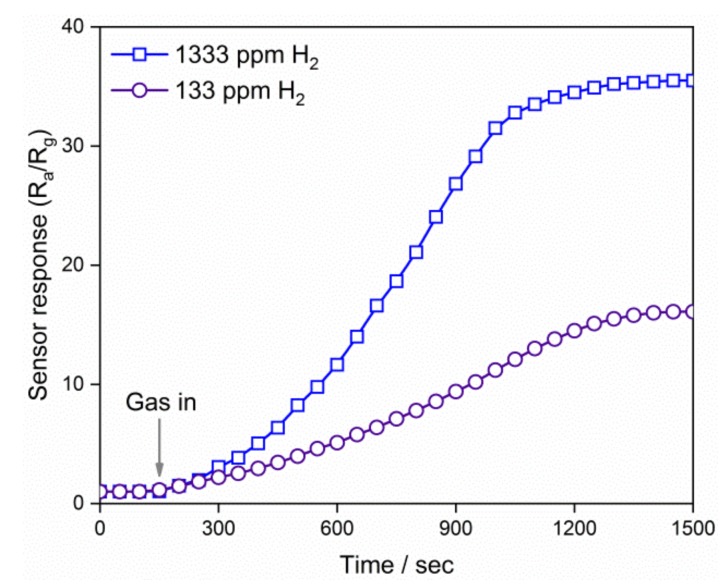
Gas-sensing characteristics of the thin film prepared from the QD0 solution at room temperature.

Figure 8 shows the dynamic response of the QD0 thin film at room temperature to the H_2_ gas with concentration of 133 and 1333 ppm, to which the responses are 16.1 and 35.5. After exposure to the reducing gas, the resistance of thin film decreased, because the reducing gas molecules consumed the adsorbed oxygen on the grain surface. The results show that the SnO_2_ gas sensor benefited from the structure of the QDs. Van Toan [40] used to fabricate SnO_2_ nanocrystalline gas sensors with grain size of 20 nm, which showed a response of 6 to 1000 ppm H_2_ at 300 °C. Kim [41] recently prepared SnO_2_ nanowire-based gas sensors, and the response of bare SnO_2_ nanowires was 8 to 100 ppm H_2_ at 300 °C. Therefore, the prepared QDs were the promising candidate for producing novel semiconductor gas sensors, which were highly sensitive at room temperature. In this work, however, only the gas of H_2_ was used to check the gas-sensing performance of QDs, as was the same operation in much other research [42,43]. It is known that the semiconductor gas sensors would show response to other gases, such as CO, NO_2_, ammonia, and acetone [44]. Thus, selectivity was one of the sensor properties that had to be concerned. The sensor properties were also found to be dependent on the characteristics of QDs, including grain size and shape [45,46,47,48]. Further investigations are expected to reveal the details of gas-sensing performances, including the grain size effect and selectivity of the semiconductor QDs.

## 4. Conclusions

The SnO_2_ QDs were prepared in the aqueous solution via a simple process of hydrolysis and oxidation. The average grain size was 1.9 nm, and it increased to 2.7 and 4.0 nm after the hydrothermal treatments at 125 and 225 °C, respectively. The rutile SnO_2_ system was confirmed by the XPS spectrum and the XRD pattern. UV-VIS absorption spectrum results had an *E_g_* value of 3.66 eV, which is a little larger than the bulk SnO_2_ system. The QDs of 0.002 mol/L showed a fluorescence emission peak at the wavelength of 300 nm, and their fluorescence response was quenched when the high concentration of 0.2 mol/L was used. The voltage-current characteristics showed that the electrical properties of the QD thin film were determined by the depletion layer, where a Schottky barrier was established at the grain boundaries. The QD thin film showed responses of 16.1 and 35.5 to H_2_ gas with concentration of 133 and 1333 ppm, respectively.

## References

[1] Maulu A., Navarro-Arenas J., Rodríguez-Cantó P.J., Sánchez-Royo J.F., Abargues R., Suárez I., Martínez-Pastor J.P. (2018). Charge transport in trap-sensitized infrared PbS quantum-dot-based photoconductors: Pros and cons. Nanomaterials.

[2] Yuan C., Li L., Huang J., Ning Z., Sun L., Ågren H. (2016). Improving the photocurrent in quantum-dot-sensitized solar cells by employing alloy Pb_x_Cd_1−x_S quantum dots as photosensitizers. Nanomaterials.

[3] Mohamed A.E., Yuangang Z., Hsiao-Hua Y., Ying J.Y. (2007). Ultrasensitive Pb^2+^ detection by glutathione-capped quantum dots. Anal. Chem..

[4] Silva do Nascimento A., Cabral Filho P.E., Fontes A., Saegesser Santos B., Rodrigues de Carvalho F., Stragevitch L., Soares Leite E. (2019). CdSe quantum dots as fluorescent nanomarkers for diesel oil. Fuel.

[5] Zhang B., Li M., Song Z., Kan H., Yu H., Liu Q., Zhang G., Liu H. (2017). Sensitive H_2_S gas sensors employing colloidal zinc oxide quantum dots. Sens. Actuators B.

[6] Liu H., Li M., Shao G., Zhang W., Wang W., Song H., Cao H., Ma W., Tang J. (2015). Enhancement of hydrogen sulfide gas sensing of PbS colloidal quantum dots by remote doping through ligand exchange. Sens. Actuators B.

[7] Li M., Luo J., Fu C., Kan H., Huang Z., Huang W., Yang S., Zhang J., Tang J., Fu Y. (2018). PbSe quantum dots-based chemiresistors for room-temperature NO_2_ detection. Sens Actuators B.

[8] Liu H., Li M., Voznyy O., Hu L., Fu Q., Zhou D., Xia Z., Sargent E.H., Tang J. (2014). Physically flexible, rapid-response gas sensor based on colloidal quantum dot solids. Adv. Mater..

[9] Song Z., Huang Z., Liu J., Hu Z., Zhang J., Zhang G., Yi F., Jiang S., Lian J., Yan J. (2018). Fully stretchable and humidity-resistant quantum dot gas sensors. ACS Sens..

[10] Zepeda A.M., Gonzalez D., Heredia L.G., Marquez K., Perez C., Pena E., Flores K., Valdes C., Eubanks T.M., Parsons J.G. (2018). Removal of Cu^2+^ and Ni^2+^ from aqueous solution using SnO_2_ nanomaterial effect of: pH, time, temperature, interfering cations. Microchem. J..

[11] Liu J., Lu Y., Cui X., Geng Y., Jin G., Zhai Z. (2017). Gas-sensing properties and sensitivity promoting mechanism of Cu-added SnO_2_ thin films deposited by ultrasonic spray pyrolysis. Sens. Actuators B.

[12] Liu J., Liu X., Zhai Z., Jin G., Jiang Q., Zhao Y., Luo C., Quan L. (2015). Evaluation of depletion layer width and gas-sensing properties of antimony-doped tin oxide thin film sensors. Sens. Actuators B.

[13] Song Z., Liu J., Liu Q., Yu H., Zhang W., Wang Y., Huang Z., Zang J., Liu H. (2017). Enhanced H_2_S gas sensing properties based on SnO_2_ quantum wire/reduced graphene oxide nanocomposites: Equilibrium and kinetics modeling. Sens. Actuators B.

[14] Liu H., Zhang W., Yu H., Liang G., Song Z., Xu S., Min L., Yang W., Song H., Jiang T. (2016). Solution-processed gas sensors employing SnO_2_ quantum dot/MWCNT Nanocomposites. ACS Appl. Mater. Interfaces.

[15] Liu J., Wang W., Zhai Z., Jin G., Chen Y., Hong W., Wu L., Gao F. (2018). Influence of oxygen vacancy behaviors in cooling process on semiconductor gas sensors: A numerical analysis. Sensors.

[16] Liu J., Wang W., Zhai Z., Jin G., Chen Y. (2018). Numerical analysis of the effect of the cooling process on the characteristics of n-type semiconductor gas sensors. J. Comput. Electron..

[17] Liu J., Gao Y., Wu X., Jin G., Zhai Z., Liu H. (2017). Inhomogeneous oxygen vacancy distribution in semiconductor gas sensors: Formation, migration and determination on gas sensing characteristics. Sensors.

[18] Zhao D., Wu X. (2018). Nanoparticles assembled SnO_2_ nanosheet photocatalysts for wastewater purification. Mater. Lett..

[19] Tammina S.K., Mandal B.K., Khan F.N. (2019). Mineralization of toxic industrial dyes by gallic acid mediated synthesized photocatalyst SnO_2_ nanoparticles. Environ. Technol. Innov..

[20] Jayaweera E.N., Kumara G.R.A., Kumarage C., Ranasinghe S.K., Rajapakse R.M.G., Bandara H.M.N., Ileperuma O.A., Dassanayake B.S. (2018). CdS nanosheet-sensitized solar cells based on SnO_2_/MgO composite films. J. Photochem. Photobiol. A.

[21] Zhang X., Rui Y., Yang J., Wang L., Wang Y., Xu J. (2019). Monodispersed SnO_2_ microspheres aggregated by tunable building units as effective photoelectrodes in solar cells. Appl. Surf. Sci..

[22] Min X., Sun B., Chen S., Fang M., Wu X., Liu Y.G., Abdelkader A., Huang Z., Liu T., Xi K. (2019). A textile-based SnO_2_ ultra-flexible electrode for lithium-ion batteries. Energy Storage Mater..

[23] Li H., Su Q., Kang J., Huang M., Feng M., Feng H., Huang P., Du G. (2018). Porous SnO_2_ hollow microspheres as anodes for high-performance lithium ion battery. Mater. Lett..

[24] Hollingsworth J.A. (2006). Semiconductor Nanocrystal Quantum Dots.

[25] Song Z., Xu S., Liu J., Hu Z., Gao N., Zhang J., Yi F., Zhang G., Jiang S., Liu H. (2018). Enhanced catalytic activity of SnO_2_ quantum dot films employing atomic ligand-exchange strategy for fast response H_2_S gas sensors. Sens. Actuators B.

[26] Liu H., Xu S., Min L., Gang S., Song H., Zhang W., Wei W., He M., Liang G., Song H. (2014). Chemiresistive gas sensors employing solution-processed metal oxide quantum dot films. Appl. Phys. Lett..

[27] Babu B., Neelakanta Reddy I., Yoo K., Kim D., Shim J. (2018). Bandgap tuning and XPS study of SnO_2_ quantum dots. Mater. Lett..

[28] Han W.-Q., Zettl A. (2003). Coating single-walled carbon nanotubes with tin oxide. Nano Lett..

[29] Cigala R.M., Crea F., de Stefano C., Lando G., Milea D., Sammartano S. (2012). The inorganic speciation of tin(II) in aqueous solution. Geochim. Cosmochim. Acta.

[30] Misra G.S., Gupta C.V. (1973). Aqueous polymerization of methacrylamide initiated by acidified bromate/thiourea redox system. Die Makromol. Chem..

[31] Swaminathan K., Irving H.M.N.H. (1964). Infra-red absorption spectra of complexes of thiourea. J. Inorg. Nucl. Chem..

[32] Seki H., Ishisawa N., Mizutani N., Kato M. (1984). High temperature structures of the rutile-type oxides, TiO_2_ and SnO_2_. J. Ceram. Assoc. Jpn..

[33] Barreca D., Garon S., Tondello E., Zanella P. (2000). SnO_2_ nanocrystalline thin films by XPS. Surf. Sci. Spectra.

[34] Stranick M.A., Moskwa A. (1993). SnO_2_ by XPS. Surf. Sci. Spectra.

[35] Song Z., Wei Z., Wang B., Zhen L., Xu S., Zhang W., Yu H., Min L., Zhao H., Zang J. (2016). Sensitive room-temperature H_2_S gas sensors employing SnO_2_ quantum wire/reduced graphene oxide nanocomposites. Chem. Mater..

[36] Henry J., Mohanraj K., Sivakumar G., Umamaheswari S. (2015). Electrochemical and fluorescence properties of SnO2 thin films and its antibacterial activity. Spectrochim. Acta Part A.

[37] Lakowicz J.R. (2006). Quenching of Fluorescence. Principles of Fluorescence Spectroscopy.

[38] Zhai D., Xu W., Zhang L., Chang Y.-T. (2014). The role of “disaggregation” in optical probe development. Chem. Soc. Rev..

[39] Gong S., Liu J., Quan L., Fu Q., Zhou D. (2011). Preparation of tin oxide thin films on silicon substrates via sol-gel routes and the prospects for the H_2_S gas sensor. Sens. Lett..

[40] Van Toan N., Viet Chien N., Van Duy N., Si Hong H., Nguyen H., Duc Hoa N., Van Hieu N. (2016). Fabrication of highly sensitive and selective H_2_ gas sensor based on SnO_2_ thin film sensitized with microsized Pd islands. J. Hazard. Mater..

[41] Kim J.-H., Mirzaei A., Kim H.W., Kim S.S. (2019). Improving the hydrogen sensing properties of SnO_2_ nanowire-based conductometric sensors by Pd-decoration. Sens. Actuators B.

[42] Sakai G., Matsunaga N., Shimanoe K., Yamazoe N. (2001). Theory of gas-diffusion controlled sensitivity for thin film semiconductor gas sensor. Sens. Actuators B.

[43] Xu C., Tamaki J., Miura N., Yamazoe N. (1991). Grain size effects on gas sensitivity of porous SnO_2_-based elements. Sens. Actuators B.

[44] Krivetskiy V., Ponzoni A., Comini E., Badalyan S., Rumyantseva M., Gaskov A. (2010). Selectivity modification of SnO_2_-based materials for gas sensor arrays. Electroanalysis.

[45] Yamazoe N., Shimanoe K. (2008). Roles of shape and size of component crystals in semiconductor gas sensors II. Response to NO_2_ and H_2_. J. Electrochem. Soc..

[46] Yamazoe N., Shimanoe K. (2008). Roles of shape and size of component crystals in semiconductor gas sensors I. Response to oxygen. J. Electrochem. Soc..

[47] Liu J., Zhai Z., Jin G., Li Y., Monica F.F., Liu X. (2015). Simulation of the grain size effect in gas-sensitive SnO_2_ thin films using the oxygen vacancy gradient distribution model. Electron. Mater. Lett..

[48] Liu J., Jin G., Zhai Z., Monica F.F., Liu X. (2015). Numeral description of grain size effects of tin oxide gas-sensitive elements and evaluation of depletion layer width. Electron. Mater. Lett..

